# A traditional evolutionary history of foot-and-mouth disease viruses in Southeast Asia challenged by analyses of non-structural protein coding sequences

**DOI:** 10.1038/s41598-018-24870-6

**Published:** 2018-04-24

**Authors:** Barbara Brito, Steven J. Pauszek, Ethan J. Hartwig, George R. Smoliga, Le T. Vu, Pham V. Dong, Carolina Stenfeldt, Luis L. Rodriguez, Donald P. King, Nick J. Knowles, Katarzyna Bachanek-Bankowska, Ngo T. Long, Do H. Dung, Jonathan Arzt

**Affiliations:** 1 Foreign Animal Disease Research Unit, Plum Island Animal Disease Center, ARS, USDA, NY, USA; 2 Oak Ridge Institute for Science and Education, PIADC Research Participation Program, Oak Ridge, TN USA; 3 Regional Animal Health Office No. 6, Department of Animal Health, Ministry of Agriculture and Rural Development, Ho Chi Minh City, Vietnam; 4 Department of Veterinary Population Medicine, College of Veterinary Medicine, University of Minnesota, St. Paul, MN USA; 5 The Pirbright Institute, Ash Road, Pirbright, Woking, Surrey, UK; 6 Department of Animal Health, Ministry of Agriculture and Rural Development, Hanoi, Vietnam

## Abstract

Recombination of rapidly evolving RNA-viruses provides an important mechanism for diversification, spread, and emergence of new variants with enhanced fitness. Foot-and-mouth disease virus (FMDV) causes an important transboundary disease of livestock that is endemic to most countries in Asia and Africa. Maintenance and spread of FMDV are driven by periods of dominance of specific viral lineages. Current understanding of the molecular epidemiology of FMDV lineages is generally based on the phylogenetic relationship of the capsid-encoding genes, with less attention to the process of recombination and evolution of non-structural proteins. In this study, the putative recombination breakpoints of FMDVs endemic to Southeast Asia were determined using full-open reading frame sequences. Subsequently, the lineages’ divergence times of recombination-free genome regions were estimated. These analyses revealed a close relationship between two of the earliest endemic viral lineages that appear unrelated when only considering the phylogeny of their capsid proteins. Contrastingly, one lineage, named O/CATHAY, known for having a particular host predilection (pigs) has evolved independently. Additionally, intra-lineage recombination occurred at different breakpoints compared to the inter-lineage process. These results provide new insights about FMDV recombination patterns and the evolutionary interdependence of FMDV serotypes and lineages.

## Introduction

Foot-and-mouth disease (FMD) is one of the most important diseases of livestock worldwide^1,2^. Many countries with endemic FMD have rural populations that are highly reliant on their livestock as critical assets. The causal agent, FMD virus (FMDV), affects cloven-hoofed animals and is endemic in all countries in mainland Southeast Asia, where clinical cases are regularly observed in livestock, including pigs, cattle, Asian buffalo and small ruminants.

FMDV belongs to the genus *Aphthovirus* of the family *Picornaviridae* and has a single-stranded, positive-sense, non-segmented RNA genome consisting of an open reading frame (ORF) region of ~7000 nucleotides (nt). The genome encodes for a single polyprotein that is post-translationally processed into 4 capsid proteins (VP1–4) and 10 non-structural proteins (NSP; leader proteinase (L^pro^), 2A, 2B, 2C, 3A, 3B1^VPg1^, 3B2^VPg2^, 3B3^VPg3^, 3C^pro^ and 3D^pol^), bounded by 5′and 3′untranslated regions (UTRs)^3^. FMDV has been classified into seven distinct serotypes, namely A, O, C, Asia-1, Southern African Territories (SAT) 1, SAT 2 and SAT 3^4^, many of which exist as multiple strains or lineages circulating in endemic regions^5^.

In general, countries that have transboundary animal trade or porous borders for animal movement also sustain the same FMDVs lineages^6^. Such regions often have similar variety and distribution of susceptible host species, husbandry practices and have frequent livestock exchange or share trading routes and markets. Currently serotypes A and O are endemic in Southeast Asia^7–10^. Serotype Asia-1 has not been reported in the region since 2009 (except for one detection in Myanmar in April 2017^11^).

Molecular epidemiology and evolution of FMDV has traditionally been studied using the sequences coding for VP1 (627–657 nt, depending on serotype), the most variable capsid structural protein containing relevant antigenic domains^12,13^. The justification for the use of VP1 is the relative ease of sequencing, high heterogeneity of the region and the reliability of virus classification. Phylogeny of VP1 correctly distinguishes each of the viral serotypes, which are further classified into topotypes (cutoff 85–80% identity of VP1 nt sequence), lineages (~90% VP1 nt identity) and sublineages (~95% VP1 nt identity), based upon VP1 phylogenetic clustering and genetic distances of the viruses and designated with an arbitrary conventional nomenclature^14–19^.

Two lineages of FMDV serotype O belonging to the SOUTHEAST ASIA (SEA) topotype circulated in Southeast Asia until the late 1990s, namely, O/Cam-94 and O/Mya-98. However, of these lineages, only O/Mya-98 is currently present and endemic to mainland countries in Southeast Asia^20,21^. Additionally, prior to 1984, two additional topotypes were present in, and probably confined to Indonesia (ISA-1 and ISA-2, now extinct); it is likely that these viruses were introduced in the late 1800s from Europe^18^. In 2010–2011 O/Mya-98 lineage spread to several Eastern Asian countries which were previously FMD-free without vaccination, including South Korea and Japan^10,22^. O/Mya-98 further diverged into two distinct sub-clusters, O/Mya-98(A) and O/Mya-98(B)^10^. Serotype O topotype CATHAY, also known as the pig-adapted FMDV, was identified for the first time in 1970 in Hong Kong SAR and China, and later spread to the Philippines (1994), Taiwan (1997), Vietnam (1997) and Malaysia (2005). Viruses belonging to this unique topotype currently circulate in Vietnam, Hong Kong SAR, China and possibly Taiwan^20,23,24^. Serotype O topotype ME-SA, lineage PanAsia (O/PanAsia) is also endemic to Southeast Asia. O/PanAsia emerged in India and spread widely in the 1990s into the Middle East and North Africa and ultimately to South Africa in 2000 and Europe in 2001. In the late 1990’s the PanAsia lineage was also introduced and spread throughout most of the countries in Southeast Asia, and has remained endemic in the region^18,20,25^.

FMDV serotype A in Southeast Asia is represented by a single lineage within the ASIA topotype named Southeast Asia-97 (A/Sea-97; also named genotype IX or 20). Serotype A in Southeast Asia was detected mostly from Malaysia and Thailand during the late 1990s, and has been more recently reported in other countries in mainland Southeast Asia and East Asia. These viruses are closely related to an FMDV from India collected in 1982^16,20^.

FMDV of the Asia-1 serotype all belong to one topotype, and have been further classified in genetic groups. Asia-1 Group IV (As1/Gr-IV) circulated in Southeast Asia at least since the 1970s, inferred by the earliest sequenced VP1s^17^. Asia-1 Group V (As1/Gr-V) emerged in China in 2005 and was also found in Mongolia, the Russian Federation, North Korea and Vietnam. This virus is related to FMDVs collected from the 1970s and 1980s in India. Finally, Asia-1 Group VI (As1/Gr-VI) was introduced into China and the Russian Federation in 2005, and was related to viruses circulating simultaneously in Pakistan, Afghanistan and Iran^17,21^.

Previous studies have reported recombination of FMDV under experimental conditions in tissue culture^26^, detection of recombination using statistical approaches to analyze FMDV sequences^27–29^, intra-typic (serotype A) capsid recombination^30^ and isolated reports of recombinant viruses found in the field^31–36^. The findings of these studies suggest that FMDV recombinations are not isolated events, but an evolutionary mechanism frequently used by FMDV, as well as other picornaviruses^37^. However, elucidation of the overall frequency of recombination and its biological significance may only become clearer as more complete genome sequences are generated and integrated with field knowledge of circulating viral lineages.

Understanding the mechanisms that drive viral diversity, and ultimately the emergence of novel, highly fit viral lineages are critical to predict patterns of FMDV occurrence and changes in seasonal or periodic dominance (lineage ‘turnover’) that are observed in endemic regions. Furthermore sporadic spillover to FMD-free countries has been closely related to dominance of highly fit viruses that were previously reported to spread widely among endemic regions similar to what occurred with the serotype O lineage PanAsia [topotype Middle East-South Asia (ME-SA)] in 2001^18^. This occurrence has been studied using structural protein-based taxonomic classification of FMDV, but the role of the evolution in distinct protein coding regions of the genome and its potential association with the dependence between lineages and serotype occurrence have not been explored. Enhanced understanding of lineage emergence and regional patterns of lineage dominance may contribute to improving disease control and preparedness.

The objective of this study was to investigate the inter- and intra-lineage recombination of the FMDV lineages that currently circulate in Southeast Asia. This study integrates knowledge of FMD molecular epidemiology and the specific implications of viral recombination. Furthermore, these results contribute to novel understanding of the evolutionary interdependence of FMDV serotypes and lineages. Such knowledge may improve FMD control through consideration of co-circulation of distinct FMDV lineages. Elucidation of the evolutionary mechanisms of FMDV may help understand and predict emergence of new lineages, and inform the risk posed by co-circulating lineages in FMD-endemic regions.

## Results

### Inter-lineage recombination

**Figure 1:**
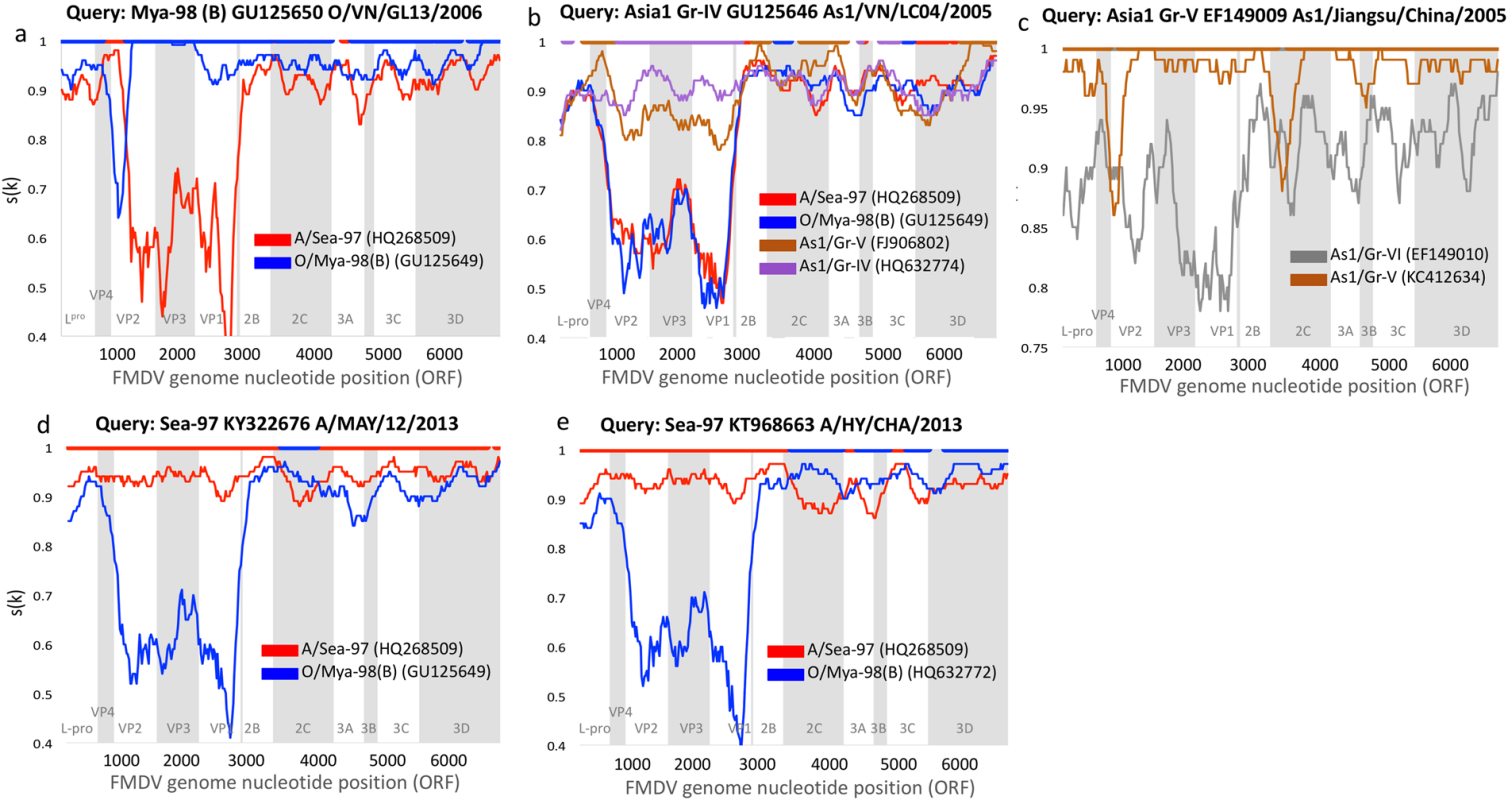
Similarity plot for unique recombination events (**a**–**e**) detected using RDP4 and phylogenetic reconstruction. The query sequence name (recombinant) is indicated at the top of each panel. The x axis indicates the nucleotide position (ORF) of the query sequence at the center of the sliding window (k). The y axis indicates the similarity, s(k), between the corresponding window of the query sequence and each of the reference sequences. A non-recombinant reference sequence from a given lineage was used to represent the relationship between the query (recombinant) sequence and each lineage. The colored bar at the top of each plot indicates the ‘best match’, which is the reference sequence with the highest identity to the query sequence. The specific reference sequence and lineages of the viruses contributing to the recombinant virus are indicated in the legend.

Using different algorithms for recombination detection, six events of unique inter-lineage viral recombinants, with identified minor and major parents, and confirmed by at least two distinct analytical techniques were detected (Table 1, Fig. 1). Event 1: a unique recent recombinant was identified in a virus from O/Mya-98(B), O/VN/GL13/2006 (GU125650) recombining with A/Sea-97 lineage (parental relationship inferred from a group of A/Sea-97 sequences), specifically at VP4 coding site (Fig. 1a). Events 2 and 3: two recombinant regions were found in the As1/Gr-IV sequence As1/VN/LC04/2005 (GU125646) within the 3D coding region (parental relationship inferred from a group of O/Mya-98 sequences) and within 2C-3A coding region (parental relationship inferred from a group of A/Sea-97 sequences; Table 1). However, this event is not clear in the similarity plot, where a higher similarity of As1/VN/LC04/2005 appears to occur with the As1/Gr-V query sequence around VP4 and through other non-structural protein (NSP) coding regions, and with A/Sea-97 in the first half of 3D (Fig. 1b). Event 4: inferred recombinant was a virus As1/Gr-V As1/Jiangsu/China/2005 (EF149009) from parental sequence As1/Gr-VI As1/HNK/CHA/05 (EF149010) high identity within the 2C coding region detected by RDP and Geneconv methods (Table 1). The similarity plot (Fig. 1c) depicts a small region within 2C and at the beginning of VP2 where recombination of these two viruses may have occurred, however the similarities observed in the plot do not provide a strong evidence to support this event. Event 5: evidence of recombination of a virus from A/Sea-97 A/MAY/12/2013 (KY322676) within the 2C coding region (parental relationship inferred from a group of O/Mya-98(B) viruses) (Fig. 1d). Event 6: Recent recombinant A/Sea-97 A/HY/CHA/2013 (KT968663), was not detected by RDP4, but was identified by the phylogenetic reconstruction of the recombination-free segments and simplot analysis that revealed high identity with lineage O/Mya-98(B) in 2C and 3C-3D coding regions (Fig. 1e).

**Table 1:** Events detected in unique recombinant viral sequences.

Event Number	Recombinant detected (lineage; virus name; GenBank accession number)	Description of recombination: protein coding region (nucleotide position in alignment of the breakpoints detected), lineage from which parental recombinant was inferred	Methods that detected recombination (P < 0.05)	Similarity plots (Fig. 1)
1	O/Mya-98(B); O/VN/GL13/2006; GU125650 (host: cattle)	Recombination at VP4 (731–1081) with lineage A/Sea-97	RDP, GC, BS, CS, CHM, SIS	Fig. 1(a)
2	As1/Gr-IV;As1/VN/LC04/2005; GU125646 (host: buffalo)	Recombination at 3D (6303–6741) with lineage O/Mya-98	RDP, GC, BS, CS, CHM	Fig. 1(b)
3	As1/Gr-IV; As1/VN/LC04/2005; GU125646 (host: buffalo)	Recombination at 2C-3A (3903–4540) with lineage A/Sea-97	RDP, GC, CS, CHM	Fig. 1(b)
4	As1-Gr-V; As1/Jiangsu/China/2005; EF149009 (host: cattle)	Recombination at 2C (3443–3587) with As1/Gr-VI As1/HNK/CHA/05 (#EF149010)	RDP, GC	Fig. 1(c)
5	A/Sea-97; A/MAY/12/2013; KY322676 (host: cattle)	Recombination at 2C (3497–4151) with lineage O/Mya-98	RDP, GC, CS, CHM	Fig. 1(d)
6	A/Sea-97; A/HY/CHA/2013; KT968663 (host: yak)	Recombination at in 2C and 3C-3D with lineage O/Mya-98(B)	Bayesian time divergence estimation	Fig. 1(e)

The events listed comprise unique sequences detected as inter-lineage recombinants with known parental sequences. The methods that significantly detected the recombination are indicated as RDP: Recombination Detection Program, GC: Geneconv, BS: Boostcan, CS: Chi square, CHM: Chimaera, SIS: Siscan or Bayesian time divergence estimation. The breakpoint position indicated is with respect the recombinant ORF sequence. The corresponding lineage and accession number of the recombinant sequence, and the parental sequence from which the recombinant region was inferred are indicated. All events are depicted in the corresponding panel of the similarity plot (Fig. 1).

### Phylogeny of FMDV recombination-free regions

**Figure 2:**
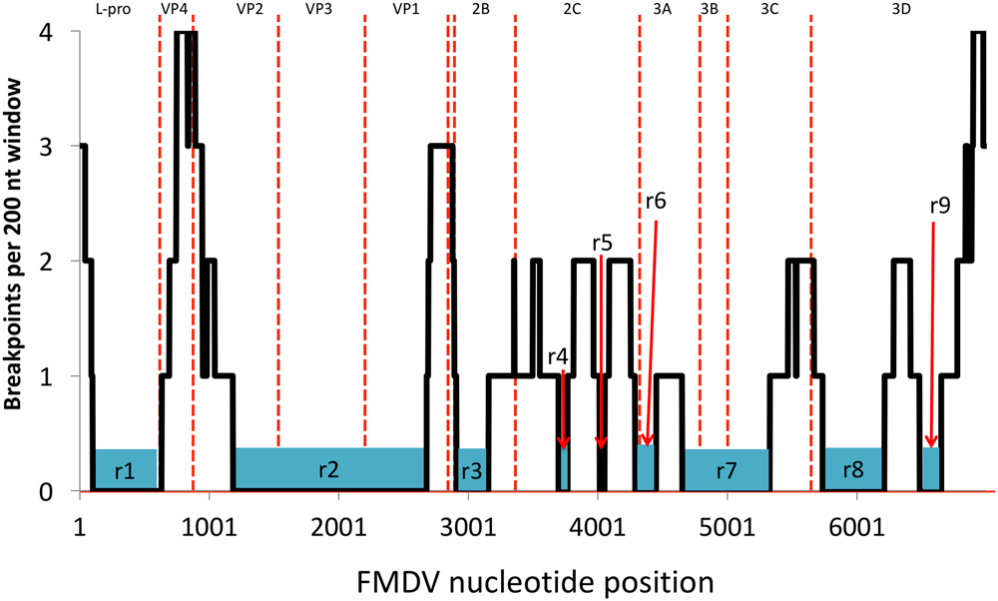
Recombination breakpoints distribution throughout ORF sequences of Southeast Asia endemic Foot-and-mouth disease virus (FMDV) lineages. Based on the recombination points found, nine recombination-free regions (r1–9) were found within the FMDV genomes analyzed. The y-axis represents the number of recombination breakpoints detected within a 300 nt sliding window. The x-axis represents the nt position across the genome (reference for nt position: sequence O/JPN/2000-AB079061).

The ancestral relationships of the viral lineages were further explored using Bayesian time divergence estimation of recombination-free regions. Using results from RDP4, a recombination distribution plot was built (Fig. 2). Based on the recombination breakpoints detected, nine different recombination-free regions were identified (defined as r1-r9). Specific regions (reference for nt positions within the ORF: sequence O/JPN/2000-AB079061) were bounded by sites 101–631 (r1), 1183–2677 (r2), 2909–3155 (r3), 3697–3765 (r4), 4013–4051 (r5), 4287–4449 (r6), 4651–5325 (r7), 5735–6211 (r8), and 6483–6649 (r9).

Likelihood mapping (percentage) of resolved phylogenies within each of the regions were r1: 7.8%, r2: 1.2%, r3: 18.8%, r4: 21.6%, r5: 50.1%, r6: 29.8%, r7: 7%, r8: 8.6%, and r9: 15.7%. On this basis, r4, r5 and r6 did not have enough signal to reconstruct the phylogenies. Bayesian phylogenetic analysis was performed for all other regions.

#### Recombination-free region r1, non-structural protein L^pro^

**Figure 3:**
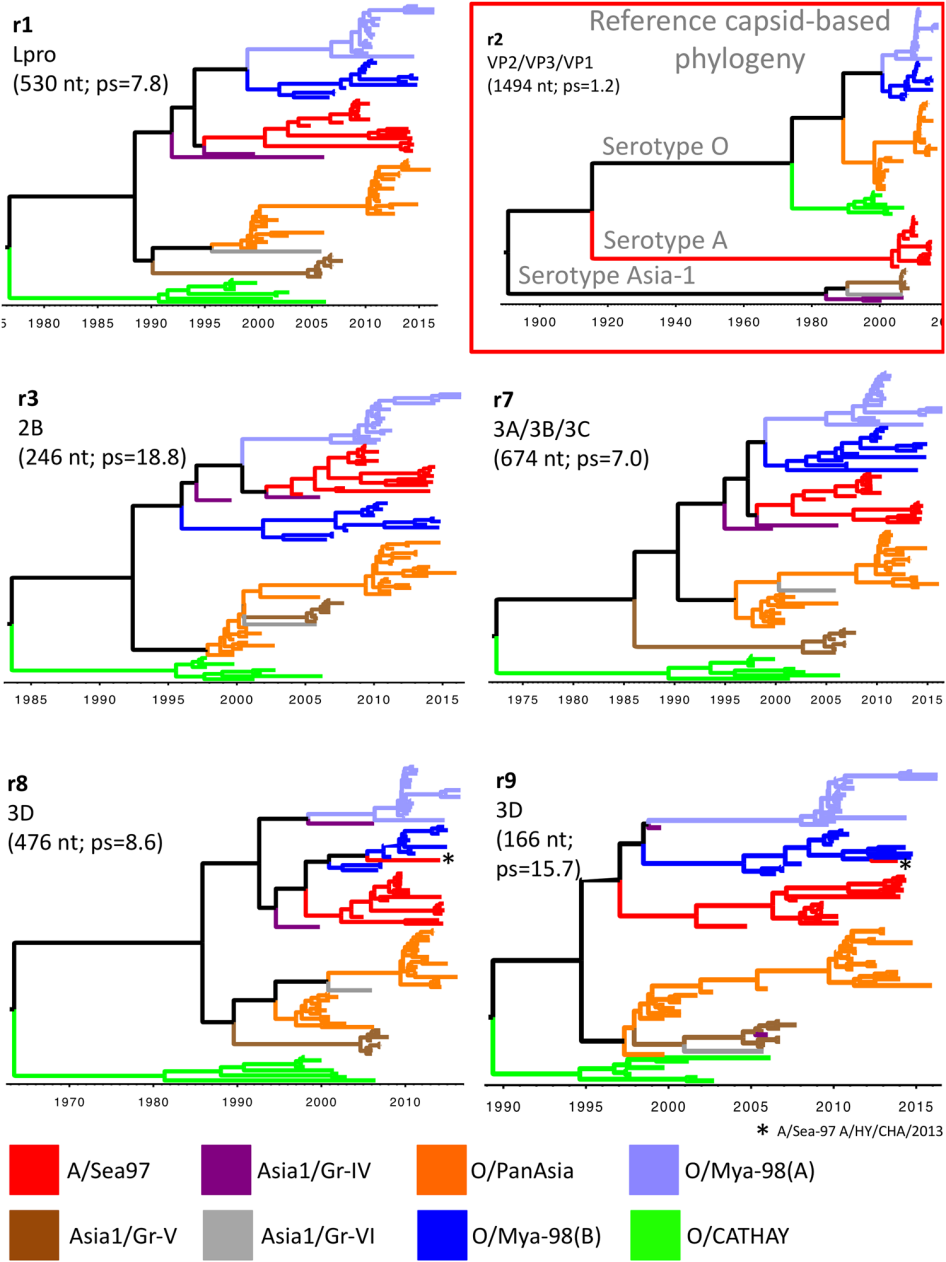
Maximum clade credibility trees constructed for recombination-free regions r1, r2, r3, r7, r8 and r9. The classification of lineages, topotypes and serotypes are defined by the traditional phylogeny grouping of viruses by their structural proteins coding regions (r2). Color-coding of the tree branches is based on this r2 classification. The topology of the phylogenetic trees based on the non-capsid coding sequence shows a close relationship between the O/Mya-98 and A/Sea-97 lineages, and an independent evolution of the viruses belonging to the O/CATHAY lineage. Recent recombinant viruses are indicated by an asterisk (*).

The r1 sequence (530 nt long) was located entirely within the L^pro^ protein-coding region. The phylogenetic topology of r1 nucleotide sequence showed a distinct clustering pattern from that observed for nucleotide sequence encoding the capsid proteins (Fig. 3). The two sublineages identified in the O/Mya-98 lineage (sublineages A and B) grouped more closely with A/Sea-97 than with other lineages, having an estimated time to most recent common ancestor (tMRCA) at 1994 (95% high posterior density (HPD) 1990–1997; Table 2). Asia-1 viruses classified within the As1/Gr-IV were related to the common ancestor of the O/Mya-98 and A/Sea-97 lineages. O/PanAsia lineage viruses were more closely related to the As1/Gr-V lineage with the tMRCA estimated to date back to 1990 (95% HPD 1984–1994). The only As1/Gr-VI virus available for analysis was closely related to the O/PanAsia ancestor. O/CATHAY viruses grouped distantly from all other lineages with the early common ancestor with all other viruses estimated to date back to 1976 (95% HPD 1964–1986).

**Table 2:** Time to most recent common ancestor (year) and 95% high posterior density (HPD) for each of the two lineages in the recombination-free regions.

Lineage name	tMRCA (95% HPD) for each pair of lineages					
	Region 1	Region 2	Region 3	Region 7	Region 8	Region 9
A/Sea-97 & O/Mya-98 (A)	1994 (1990, 1997)	1914 (1866, 1951)	2000 (1997, 2003)	1997 (1993, 2000)	1992 (1987, 1996)	1997 (1993, 1999)
A/Sea-97 & O/Mya-98 (B)	1994 (1990, 1997)	1914 (1866, 1951)	1996 (1992, 1999)	1997 (1993, 2000)	1998 (1994, 2001)	1997 (1993, 1999)
A/Sea-97 & O/ PanAsia	1988 (1982, 1993)	1914 (1866, 1951)	1992 (1987, 1996)	1990 (1984, 1995)	1985 (1976, 1992)	1994 (1989, 1998)
A/Sea-97 & As1/Gr-V	1988 (1982, 1993)	1889 (1832, 1937)	1992 (1987, 1996)	1986 (1977, 1993)	1985 (1976, 1992)	1994 (1989, 1998)
A/Sea-97 & O/CATHAY	1976 (1964, 1986)	1914 (1866, 1951)	1983 (1974, 1990)	1972 (1953, 1985)	1963 (1938,1981)	1989 (1980, 1995)
O/Mya-98 (A) & O/Mya-98 (B)	1999 (1995, 2002)	2000 (1996, 2002)	1996 (1992, 1999)	1999 (1995, 2002)	1992 (1987, 1996)	1998 (1996, 1999)
O/Mya-98 (A) & O/PanAsia	1988 (1982, 1993)	1988 (1980, 1994)	1992 (1987, 1996)	1990 (1984, 1995)	1985 (1976, 1992)	1994 (1989, 1998)
O/Mya-98 (A) & As1/Gr-V	1988 (1982, 1993)	1889 (1832, 1937)	1992 (1987, 1996)	1986 (1977, 1993)	1985 (1976, 1992)	1994 (1989, 1998)
O/Mya-98 (A) & O/CATHAY	1976 (1964, 1986)	1973 (1960, 1984)	1983 (1974, 1990)	1972 (1953, 1985)	1963 (1938, 1981)	1989 (1980, 1995)
O/Mya-98 (B) & O/PanAsia	1988 (1982, 1993)	1988 (1980, 1994)	1992 (1987, 1996)	1990 (1984, 1995)	1985 (1976, 1992)	1994 (1989, 1998)
O/Mya-98 (B) & As1/Gr-V	1988 (1982, 1993)	1889 (1832, 1937)	1992 (1987, 1996)	1986 (1977, 1993)	1985 (1976, 1992)	1994 (1989, 1998)
O/Mya-98 (B) & O/CATHAY	1976 (1964, 1986)	1973 (1960, 1984)	1983 (1974, 1990)	1972 (1953, 1985)	1963 (1938, 1981)	1989 (1980, 1995)
O/PanAsia & As1/Gr-V	1990 (1984, 1994)	1889 (1832, 1937)	2000 (1997, 2003)	1986 (1977, 1993)	1989 (1981, 1994)	1998 (1996, 1999)
O/PanAsia & O/CATHAY	1976 (1964, 1986)	1973 (1960, 1984)	1983 (1974, 1990)	1972 (1953, 1985)	1963 (1938, 1981)	1989 (1980, 1995)
As1/Gr-V & O/CATHAY	1976 (1964, 1986)	1889 (1832, 1937)	1983 (1974, 1990)	1972 (1953, 1985)	1963 (1938, 1981)	1989 (1980, 1995)

#### Recombination-free region r2, capsid proteins VP2, VP3 and VP1

Region r2 (1494 nt long) contained most of the structural proteins including partial VP2, complete VP3 and partial VP1 coding regions (Fig. 3). As expected, the traditional grouping of serotypes, topotypes, and lineages were clearly reproduced by the phylogeny of this region. There was an early time of divergence between viruses of serotype Asia-1 and serotypes A and O in 1889 (95% HPD 1832–1937), and a divergence between A and O serotypes in 1914 (95% HPD 1866–1951; Table 1). Divergence between O/CATHAY topotype and other serotype O viruses was estimated at 1976 (95% HPD 1964–1986). Divergence between serotype O ME-SA topotype and O SEA topotype was estimated at 1988 (95% HPD 1980–1994). Lastly, divergence between O/Mya-98 (A) and (B) was estimated at 2000 (95% HPD 1996–2002).

#### Recombination-free region r3, non-structural protein 2B

Overall tMRCA estimated for r3 (246 nt long), encoding partial 2B, were more recent than the ones estimated by other regions, however, the grouping pattern had a similar topology as exhibited by other NSP coding regions (Fig. 3). There was a close relationship between viruses from the O/Mya-98(A) group and A/Sea-97 2000 (95% HPD 1997–2003). Similar to the r1 topology, the two viruses from As1/Gr-IV were closely related with the Southeast Asia-endemic A/Sea-97 and O/Mya-98(A) lineages. O/Mya-98(B) clustered with A/Sea-97 and O/Mya-98(A) viruses within this region. The phylogeny of this region suggests a recombination event in 2000 (95% HPD 1997–2003) between viruses from As1/Gr-V lineage and O/PanAsia viruses collected from the early 2000s resulting in the O/PanAsia viruses collected from Southeast Asia after 2010. Similar to results for the r1 region results, O/CATHAY was the most divergent, independently evolving lineage (tMRCA 1983 95% HPD 1974–1990).

#### Recombination-free region r7, non-structural proteins 3A/3B/3C

The s7 region (674 nt long) spanned the 3′ end of 3A, all of 3B and most of 3C protein-coding regions. Similar to other NSP coding regions, the phylogeny of this region demonstrated a close relationship between O/Mya-98 (A-B) and A/Sea97 lineages, including a common ancestor estimated at 1997 (95% HPD 1993–2000). Consistent with other NSP tree topology, the two As1/Gr-IV viruses were related to O/Mya-98 and A/Sea-97 ancestors. In contrast to other phylogenies, the r7 region of As1/Gr-V lineage was divergent from O/PanAsia and the A/Sea-97-O/Mya-98 groups, having an earlier ancestor at 1986 (95% HPD 1977–1993). O/PanAsia viruses collected after 2010 had a common ancestor with the As1/Gr-VI virus. This region of O/CATHAY topotype evolved independently since 1972 (95% HPD 1953–1985).

#### Recombination-free region r8, non-structural protein 3D

Region r8 (476 nt long) was entirely within the 3D protein-coding region. For this region, A/Sea-97 viruses had a recent common ancestor with viruses from the O/Mya-98(B) lineages (tMRCA 1998 95% HPD 1994–2001). A/Sea-97 recent recombinant virus A/HY/CHA/2013 (KT968663) clustered within the O/Mya-98(B) lineage. Clustering of O/PanAsia and Asia-1 viruses was similar to r1 (L^pro^) with an estimated common ancestor at 1989 (95% HPD 1981–1994). O/CATHAY virus evolved independently from the other lineages analyzed with tMRCA of 1963 (95% HPD 1939–1981).

#### Recombination-free region r9, non-structural protein 3D

Region r9 (166 nt long) covered a small fraction of the 3D coding region. The topology of this region was similar to other NSP coding regions; A/Sea-97 viruses are related to O/Mya-98 (A-B) having a common ancestor estimated at 1997 (95% HPD 1993–1999). The same A/Sea-97 recent recombinant within r8 was detected among the O/Mya-98(B) viruses (A/HY/CHA/2013; KT968663). Interestingly, one of the As1/Gr-IV virus (As1/VN/LC04/2005; GU125646), of which all other NSP coding regions were associated with O/Mya-98, and A/Sea-97 viruses, was closely related to the As1/Gr-V viruses in r9 (Fig. 3). The relationship of As1/Gr-IV with Gr-V virus is also evident in the similarity plot (Fig. 1b), specifically in the end of 3D coding region. Similar to other NSP coding regions, O/PanAsia viruses were more closely associated with As1/Gr-V viruses. O/CATHAY was divergent from other viruses, with a common ancestor of r9 estimated at 1989 (95% HPD 1980–1995).

### Mean substitution rates

The molecular clock was computed for each recombination-free genome region and for each lineage (Table 3). As expected, region r2 (structural proteins coding region) had the highest overall substitution rate across all lineages 6.52 × 10^−3^ (95% HPD 4.85–8.40 × 10^−3^) compared to all other regions. The second highest substation rate occurred in region r1, L^pro^ (5.88 × 10^−3^ −95% HPD 4.70–7.08 × 10^−3^) followed by region r9 3D 5.44 × 10^−3^ (95% HPD 3.54–7.32 × 10^−3^). Lower substitution rates were estimated for other NSP coding regions r3 (2B) and r8 (3D) with a mean rate of 5.02 × 10^−3^ (95% HPD 3.47–6.64 × 10^−3^) and 5.00 × 10^−3^ (95% HPD 3.60–6.44 × 10^−3^) respectively. Region r7 (3A-3C) had a lower substitution rate compared to all other regions estimated at 4.29 × 10^−3^ (95% HPD 3.22 × 10^−3^, 5.38 × 10^−3^).

**Table 3:** Mean nt substitution rate (nt/site/year) and 95% HPD of each of the genome regions.

	Mean nucleotide/substitution/site/year (95% HPD)					
	Region 1	Region 2	Region 3	Region 7	Region 8*	Region 9*
A/Sea-97	6.29 × 10^−3^	6.58 × 10^−3^	5.04 × 10^−3^	4.96 × 10^−3^	5.58 × 10^−3^	5.82 × 10^−3^
	(4.84 × 10^−3^, 7.81 × 10^−3^)	(5.07 × 10^−3^, 8.18 × 10^−3^)	(3.48 × 10^−3^, 6.72 × 10^−3^)	(3.73 × 10^−3^, 6.39 × 10^−3^)	(4.01 × 10^−3^, 7.28 × 10^−3^)	(3.70 × 10^−3^, 8.0354 × 10^−3^)
O/Mya-98(A)	6.31 × 10^−3^	6.26 × 10^−3^	5.00 × 10^−3^	4.78 × 10^−3^	5.56 × 10^−3^	5.72 × 10^−3^
	(4.78 × 10^−3^, 7.81 × 10^−3^)	(4.92 × 10^−3^, 7.72 × 10^−3^)	(3.49 × 10^−3^, 6.64 × 10^−3^)	(3.34 × 10^−3^, 6.30 × 10^−3^)	(3.80 × 10^−3^, 7.30 × 10^−3^)	(3.57 × 10^−3^, 8.25 × 10^−3^)
O/Mya-98(B)	6.41 × 10^−3^	6.18 × 10^−3^	5.07 × 10^−3^	4.90 × 10^−3^	5.47 × 10^−3^	5.99 × 10^−3^
	(4.86 × 10^−3^, 8.076 × 10^−3^)	(4.78 × 10^−3^, 7.61 × 10^−3^)	(3.51 × 10^−3^, 6.82 × 10^−3^)	(3.40 × 10^−3^, 6.60 × 10^−3^)	(3.72 × 10^−3^, 7.33 × 10^−3^)	(3.59 × 10^−3^, 8.65 × 10^−3^)
O/PanAsia	6.09 × 10^−3^	6.35 × 10^−3^	5.03 × 10^−3^	4.82 × 10^−3^	5.28 × 10^−3^	5.59 × 10^−3^
	(4.81 × 10^−3^, 7.44 × 10^−3^)	(5.17 × 10^−3^, 7.63 × 10^−3^)	(3.51 × 10^−3^, 6.70 × 10^−3^)	(3.58 × 10^−3^, 6.23 × 10^−3^)	(3.80 × 10^−3^, 6.93 × 10^−3^)	(3.55 × 10^−3^, 7.75 × 10^−3^)
As1/Gr-V	5.66 × 10^−3^	5.40 × 10^−3^	4.99 × 10^−3^	3.88 × 10^−3^	4.64 × 10^−3^	5.35 × 10^−3^
	(4.03 × 10^−3^, 7.46 × 10^−3^)	(3.79 × 10^−3^, 6.96 × 10^−3^	(3.38 × 10^−3^, 6.67 × 10^−3^)	(2.40 × 10^−3^, 5.40 × 10^−3^)	(2.85 × 10^−3^, 6.50 × 10^−3^)	(2.99 × 10^−3^, 7.91 × 10^−3^)
O/CATHAY	5.94 × 10^−3^	5.64 × 10^−3^	5.05 × 10^−3^	4.46 × 10^−3^	5.13 × 10^−3^	5.63 × 10^−3^
	(4.20 × 10^−3^, 7.85 × 10^−3^)	(4.04 × 10^−3^, 7.32 × 10^−3^)	(3.42 × 10^−3^, 6.79 × 10^−3^)	(2.73 × 10^−3^, 6.19 × 10^−3^)	(3.10 × 10^−3^, 7.12 × 10^−3^)	(3.24 × 10^−3^, 8.20 × 10^−3^)
All lineages	5.88 × 10^−3^	6.52 × 10^−3^	5.02 × 10^−3^	4.29 × 10^−3^	5.00 × 10^−3^	5.44 × 10^−3^
	(4.70 × 10^−3^, 7.08 × 10^−3^)	(4.85 × 10^−3^, 8.40 × 10^−3^)	(3.47 × 10^−3^, 6.64 × 10^−3^)	(3.22 × 10^−3^, 5.38 × 10^−3^)	(3.60 × 10^−3^, 6.44 × 10^−3^)	(3.54 × 10^−3^, 7.32 × 10^−3^)

For regions 8 and 9, the sequence KT968663 (A/HY/CHA/2013) was included in the monophyletic clade of Mya-98(B) lineage for the computation of the clade specific rate.

In general, a slightly lower rate was calculated for As1/Gr-V viruses followed by CATHAY in r1, r2, r7 and r8, while very similar distribution of substitution rates in all lineages were estimated for r3 (Table 3).

### Intra-lineage recombination

**Figure 4:**
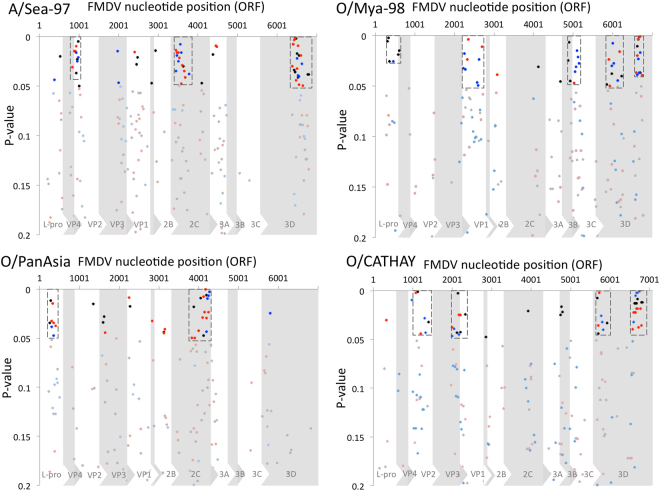
Within-lineage recombination breakpoints detected by Homoplasy tests (lineages A/Sea-97, O/Mya-98, O/PanAsia, O/CATHAY). P-values < 0.2 are plotted at the center of the window tested. Results are combined for the analyses using window sizes 300 (grey/black), 400 (pink/red) and 600 (light blue/blue). Brighter colors (black, red, blue) show the center of the window for p-value < 0.05. Specific areas with a strong statistical evidence of recombination (clustered p-values < 0.05) are shown in a dashed rectangle.

The computed mean within-lineage p-distance for the sequences coding the ORF was 0.043 for O/PanAsia, 0.046 for A/Sea97, 0.029 for O/Mya-98, and 0.020 for O/CATHAY. The plots of the homoplasy test indicate different hotspots of recombination within lineages A/Sea97, O/Mya98, O/PanAsia, and O/CATHAY (Fig. 4). For viruses within the A/Sea-97 lineage, strong evidence of recombination was identified, centered upon three specific regions bounded by nt sites: ~825–1000 mostly in VP2 coding region, ~3450–3775 within 2C coding region, and ~6375–6800 within 3D coding region. O/Mya98 lineage had at least five areas with evidence of within-lineage recombination centered upon nt sites: ~300–600 in L^pro^, ~2225–3075 mostly in VP1, ~4675–5150 in 3A, 3B, 3C, ~5850–6225 in 3D, and ~6600–6750 in 3D. O/PanAsia lineage had two evident clusters of within-lineage recombination at ~250–400 in L^pro^, and ~3825–4275 in 2C. Four main recombination hotspots were detected in O/CATHAY sequences, located between nt regions ~975–1400 in VP4-VP2, ~2000–2300 mostly within VP3, ~5700–5900 in 3D, and ~6600–6800 within 3D.

Additionally, lineages O/PanAsia and A/Sea-97 had scattered and less significant evidence of recombinant points within the capsid protein, whereas recombination within structural proteins coding regions was more significant across O/Mya-98 (VP1 and between VP3 and VP1) and in O/CATHAY viruses (between VP3 and VP1; Fig. 4). Recombination breakpoints within 3D were highly supported for lineage A/Sea-97, O/Mya-98 and O/CATHAY.

## Discussion

In this study, we have characterized the ancestral relationships between the viral lineages endemic to Southeast Asia using full ORF sequences. These analyses indicate that recombination plays an important role in FMDV evolution, and provides a new perspective of the importance of the co-circulation of different lineages and serotypes in endemic regions.

This study provides evidence for genetic association of the NSP coding regions between the endemic lineages that co-circulate within Southeast Asia. The most apparent association was found between O/Mya-98 and A/Sea-97 viruses, which have been endemic to Southeast Asia for decades, and their NSP are genetically closely related. Furthermore, the three recent recombinant viruses identified in this study are a result of exchange of genomic sequences between these two lineages (A/Sea-97 and O/Mya-98). Viruses from As1/Gr-IV also shared recent common ancestors with O/Mya-98 and A/Sea-97 in most of their NSP genomic regions. As1/Gr-IV has a long history of endemic circulation in Southeast Asia^17^, which may have allowed the exchange of NSP coding regions with the endemic serotypes A and O. However, a more detailed relationship with Asia-1 is limited by the few full ORF sequences available for analyses.

Another interesting aspect of the endemic lineage O/Mya-98, which had previously been classified into two different sublineages: A and B (based upon VP1 coding region), is the evolutionary process inferred by the analyses of the NSP coding regions^10^. Using ORF sequences, we determined that these viral sublineages were not only divergent in their structural proteins but also had distinct ancestral relationships in their NSP regions. r1 (L^pro^), r7 (3 A, 3B, and 3 C), and r9 (3D) had a remarkable consistency with Mya-98 A-B capsid-based time divergence estimates of 1999–2000, while r3 and r8 phylogenies yielded an even earlier divergence time. The initial recombination event in these regions (r3 and r8) may have triggered the divergence of the two lineages, later evidenced by the differences in the capsid coding regions. However, it is not possible to conclude this hypothesis without further field and experimental evidence.

By contrast, O/PanAsia and As1/Gr-V viruses have been endemic to Southeast Asia only since approximately 2000 and 2005, respectively, and no recombinants between these viruses and the earlier Southeast Asia endemic lineages were detected. A study which included analysis of 12 representative viruses of serotype O and Asia-1 had previously suggested recombination inferred by a higher identity of NSP, as well as the 5′ UTR and 3′ UTR between an O/PanAsia and an As1/Gr-V virus from China^32^. Using a combination of recombination detection analysis and Bayesian time divergence estimation, we were able to further characterize the ancestral relationship between O/PanAsia and As1/Gr-V and a potential more recent recombination event in 2B. Common recent ancestors of O/PanAsia and As1/Gr-V and possible recombination events are consistent with the historical geographic circulation of these viruses. O/PanAsia lineage emerged in India during the 1980s, whereas known circulation of As1/Gr-V can also be traced to India at similar times (1976–1981)^17,18,25^. Only one As1/Gr-VI full genome sequence was available, which NSP were closely related to O/PanAsia lineage. However, because only one viral sequence from this lineage was included, it is difficult to infer if this was a result of an isolated recombination event, or if this ancestral association was imprinted in all As1/Gr-VI lineage descendants.

Overall, these results indicate that recombination may not be equally likely between all FMDV lineages. Copy-choice recombination is the most likely mechanism used by FMDV, and it requires the RNA polymerase to switch between the RNA molecule templates guided by sequence similarities. Critical genetic similarities between NSP may be necessary for copy-choice occurring between certain lineages. This study suggest that these similarities may be shared by the O/Mya-98-A/Sea-97-As1/G-IV groups, and the O/PanAsia-As1/G-V groups^38^ although further field and experimental data may be needed to support this hypothesis.

The CATHAY topotype was first identified in 1970 in Hong Kong pig farms and subsequently spread sporadically into several Southeast Asian countries^20,24^. Divergence between CATHAY topotype and other serotype O viruses within capsid-coding regions was estimated at 1973 (95% HPD 1960–1984), close to the period when this topotype was first described. Divergence inferred by NSP coding regions was estimated at similar tMRCA times (r1, 1976; r3, 1983; r7, 1972; r8, 1963, and r9, 1989). Phylogenetic analyses of all regions are consistent in suggesting that CATHAY viruses evolved independently from all other co-circulating lineages, as previously suggested^29^. The lack of recombination with other picornaviruses from the same species can result in the emergence of a new species or critical deterioration of the viral sequences, as previously proposed^37^. However, neither of these evolutionary theories may be resolved by the analyses described herein. Further analyses may be facilitated by acquisition of additional sequences of CATHAY strains of FMDV.

Intra-lineage recombination is more difficult to detect compared to recombination across distant viral lineages. A previous study of O/PanAsia lineage using scanning windows did not detect intra-lineage recombination^39^, in contrast with the results obtained herein. Because of the high nt identity within FMDV lineages, methods that have a higher sensitivity to detect recombination may be useful to reveal the evolutionary association within FMDVs lineages^40^. Detection of recombination among highly homologous viruses is challenging due to the analytic limitations in differentiating recombination from point mutations. This detection becomes even more complex when considering the principles of clonal interference, competition of mutants, and quasispecies. Additionally, the biological significance of intra-lineage recombination may be different from that occurring between lineages. It has been described that such differences may be related to the distinction between short geo-temporal scale (intra-lineage) versus longer and more regional or global scales of recombination (inter-lineage)^41^. Furthermore, it has also been suggested that for rapidly evolving RNA viruses, recombination in a short time scale is a mechanism that promotes viral diversity and through which deleterious mutations are purged^37,42^. Inter-lineage recombination, by contrast, may contribute to confer critical changes that dramatically improve the fitness of the virus, as well as maintaining a stable global gene pool of a species^37^.

Although recombination of positive-sense RNA viruses is a well-established fact^27,40^, the mechanisms that enable these events at the cellular, host organism, and population levels are poorly understood. Recombination is believed to be dependent upon simultaneous replication of distinct viruses within the same cell, co-occupying the same replication complex. Numerous studies, especially in African countries have reported serological evidence of individual animals infected by multiple FMDV serotypes^43–48^, which highlights the common occurrence of multiple serotypes affecting herds in endemic countries. Additionally, one study in Pakistan, demonstrated detection and sequencing of two viruses from one sample with different serotypes (A and Asia-1), and another sample with two different sublineages of A/Iran-05^49^. Hypothetically, co-infection might occur during either the acute or post-acute phases of FMDV infection, each of which presents unique mechanistic challenges. The acute phase of FMD is relatively short, lasting 5–10 days^4,50^. The extremely high levels of virus replication at lesion sites would favor recombination; however, the activation of the innate immune response during early infection would highly impede a second strain from superinfecting an animal during the acute phase of infection. Alternatively, recombination could occur during the post-acute convalescent or carrier stages of dual infections that can last for months-years^51,52^. Heterologous co-infection in this period would be favored by the lack of innate, pro-inflammatory immune responses^53^, and absence of heterologous adaptive immune responses^54^. However, relatively low levels of viral replication and the limited anatomic distribution of infection sites during the carrier stage would not favor such events. Overall, we support the hypothesis that recombination would be most likely when a post-acute, carrier ruminant becomes superinfected with a heterologous strain and develops acute FMD. This scenario would preclude the interference of innate and adaptive immunity and would include the high levels of replication and viremia of the superinfecting virus. Furthermore, we propose that this recombination would be most likely to occur in the nasopharyngeal epithelial cells where FMDV has been demonstrated to establish and maintain persistent infection^55,56^. This proposed localization of recombination to the nasopharyngeal cells of ruminants is supported by the finding that the lineage which has evolved most independently is O/CATHAY which is more associated with clinical disease in pigs (that do not develop persistent infection), and less reported in cattle^57,58^.

The high frequency of picornaviruses recombination highlights the importance testing for recombination before reconstructing FMDV phylogenies. Most molecular epidemiologic studies of FMDV have been performed using phylogenies of VP1 or P1 regions. Under such conditions, recombination may not have greatly interfered with the assumptions. However, as whole genome sequence becomes more readily available for data analysis, assessment of recombination should always be considered as a factor that may unduly bias phylogenetic analyses and the dating of ancestral intermediates^36^.

In conclusion, we have identified novel evolutionary events within a limited set of full ORF sequences of FMDV field strains from Southeast Asia. Knowledge of viral circulation in Southeast Asia, recombination detection, and time divergence estimation were used to determine the unique evolutionary relationships amongst these viruses. Intra-lineage recombination was shown to be a recurring component of FMDV evolution, however, further analyses are required to acquire a deeper understanding of its biological significance. Inter-lineage recombination occurs in viruses with higher homologous NSP coding regions and seems to follow patterns defined by historical co-circulation. Overall, enhanced knowledge of the specific functions of the NSP and their contributions to viral fitness changes acquired by recombination may contribute to greater understanding of how specific events lead to outcompeting strains. Elucidation and assimilation of the specific mechanisms of viral infection at the molecular, host organism, and population levels will be critical to achieving the ultimate goal of understanding and predicting the emergence of novel FMDV strains with regional and pandemic spread potential. The continuous development of sequencing technologies and associated analytic tools have the potential to allow for full genome sequencing of FMDVs to be used as a routine surveillance tool which will further enable similar analyses and provide greater insights to FMDV evolution under natural conditions.

## Methods

### Data source

FMDV sequences available in GenBank public database were included in the analyses described herein. Viral sequences in GenBank were located searching the term ‘Foot-and-mouth disease virus’ (organism) and filtered by nt length >6000 to include all potential sequences of full or near full genome. Additionally, 18 FMDV sequences were obtained from samples collected and sequenced for this study at Plum Island Animal Disease Center, NY, USA. These samples were obtained from lesions in clinically affected animals during FMD outbreaks and from persistently infected animals collected in Vietnam. Further 10 sequences were obtained from samples held at the repository of the World Reference Laboratory for FMD (WRLFMD), Pirbright, United Kingdom. The lineages of all sequences of viruses described herein were identified following phylogenetic comparisons of the VP1 coding region and determined to belong to Southeast Asia endemic lineages A/Sea-97 (n = 17), O/PanAsia (n = 29), O/Mya-98 (n = 30), O/CATHAY, (n = 8), Asia-1(As1)/Gr-V (n = 8), As1/Gr-IV (n = 2) or As1/Gr-VI (n = 1), based upon VP1 protein coding region phylogenic classification (Table S1).

### Viral sequencing

At PIADC, Sanger sequencing of the full open reading frame (ORF) was performed according to a previously described method^59^. Briefly, complete bidirectional ORF sequences were obtained by amplifying each FMDV with three sets of primers. These overlapping fragments of approximately 3000 nt in length were subsequently bidirectionally sequenced by several specifically designed primers spanning the whole ORF. At PIADC, NGS sequencing of the full ORF was also performed according to a previously described method^60^. To accommodate running the SISPA-generated libraries on an Illumina MiSeq, New England Biolabs (NEB), end prep and ligation modules (E7546S, E7595S) with Bio Scientific barcoded adapters (514113) were used. ORF consensus sequences were generated after mapping assembly of the reads to the closest reference genome available in public databases followed by de novo assembly of the mapped reads. Near-complete genome sequences at The Pirbright Institute were determined as previously described using an Illumina MiSeq.^61^ and assembly of raw paired-end reads to consensus-level sequences was undertaken using SeqMan NGen® and SeqMan Pro™ (Lasergene package version 12; DNAStar, Inc.).

### Sequence alignment

The full ORF polyprotein-coding genetic region was analyzed. To validate the alignment, each of the protein coding region nt sequences (VP4–1, 2A, 2B, 2C, 3A, 3B, 3C and 3D) were aligned separately using MUSCLE, and subsequently concatenated. Because of the high heterogeneity among VP1 protein coding sequence of viruses from different serotypes, the amino acid translated sequences were aligned and then back translated into nt sequences in MEGA7^62^.

### Inter-lineage recombination identification of recombination breakpoints

Recombination analysis of 96 complete ORF sequences was carried out using RDP4 software^63^. We set the parameters to identify recombination events with multiple comparison correction (Bonferroni) and a 0.05 significance. The following algorithms implemented within RDP4 were used (1) RDP using window size = 40 (2) Geneconv (default settings) (3) Bootscan using window size = 300, step size = 20, bootstrap = 100, and Neighbor-Joining tree with a Jin and Nei model option. (4) Chi square using variable sites per window = 60 (5) Chimaera using variable sites per window = 60 and (6) Siscan using window size = 300. This analysis allowed us to (1) identify and characterize unique recombinants, which were further analyzed using a similarity plot implemented in Simplot 3.5.1^64^, where non-recombinant reference sequences from each lineage was used to represent the relationship between the query (recombinant) sequence and each lineage. (2) identify recombinant lineage (when parental recombination regions were inferred for more than one sequence from the same lineage), and (3) build a breakpoint recombination distribution plot to identify recombination-free regions using a window size of 300 nt.

### Phylogenetic analysis of recombination-free regions

A recombination breakpoints distribution plot (with window size = 200 nt) was constructed to identify the regions within the genome where recombination was not present. The recombination-free regions were obtained as separate alignments. Likelihood-mapping algorithm implemented in TreePuzzle^65^ was used to determine if the phylogenetic signal contained in each of the recombination-free regions was appropriate to reconstruct the phylogeny. Briefly, the adequacy of phylogenetic signal was defined when >80% of all possible three trees constructed by any four sequences in the alignments were resolved based on the support of the internal tree branches.

Phylogenies and divergence time estimation of FMDV using regions free of recombination with adequate phylogenetic signal were reconstructed using a Bayesian framework implemented in BEAST 1.8.4^66^. A lognormal uncorrelated relaxed clock, a Bayesian skyline tree prior and the GTR + I + G nucleotide substitution model were used for all recombination-free regions. Analyses were run for 10^8^ iterations or until all parameters reached an effective sample size >200. Mixing and convergence of the chains was assessed using Tracer v1.6^67^. The maximum clade credibility (MCC) tree was depicted in Figtree 1.4.3^68^.

The time to most recent common ancestor (tMRCA) and 95% high posterior density (HPD) of different lineages in each of the regions was extracted from annotated MCC tree to determine the evolutionary relationship of the different lineages. All viruses from serotype O, A and Asia-1 (As1) across regions were named according to their VP1 lineage classification (conventionally named O/Mya-98, O/PanAsia, O/CATHAY, A/Sea-97, As1/Group(Gr)-V, As1/Gr-IV, As1/Gr-VI). If any of the sequences in NSP coding regions clustered with a different lineage monophyletic group, it was considered an inter-lineage ‘recent recombinant’, and analysis of specific recombination was further explored using Simplot with a 200–300 nt sliding window. Bayesian phylogenetic analyses were run using computational resources available in CIPRES^69^. Genetic distances of nucleotide sequences were computed using the Kimura-2 parameters substitution using MEGA7^62^.

### Intra-lineage recombination

The intra-lineage mean pairwise genetic p-distance for full ORF was computed using MEGA7^62^. We used the Homoplasy test, which has a higher sensitivity to detect recombination in alignments with high nt identity (such as the intra-lineage ORF identity)^70^. This test accounts for the substitution patterns and rate of variation among sites to increase power and avoid false positives generated by high rates of variation across sites usually observed in virus evolution^40^. A ‘homoplasy ratio’ is calculated which ranges from zero (clonal population) to one (population under free recombination). We computed the Pairwise Homoplasy Index tests using the algorithm developed in PhiPack^70^. We ran the analyses for lineages currently present in SEA: A/Sea-97, O/PanAsia, O/Mya-98 and O/CATHAY using different window sizes of 300, 400 and 600 nt in steps of 25. We computed and plotted the specific p-values (null hypothesis of no recombination) along the viral genome to determine potential intra-lineage recombination regions.

## Electronic supplementary material

Supplementary Table 1
