# Supplementary material for: A traditional evolutionary history of foot-and-mouth disease viruses in Southeast Asia challenged by analyses of non-structural protein coding sequences
Source: Sci Rep. 2018 Apr 24;8:6472. doi: 10.1038/s41598-018-24870-6 (PMC5915611; doi:10.1038/s41598-018-24870-6)
Supplement: Supplementary file 1 — Supplementary Table 1 [file 41598_2018_24870_MOESM1_ESM.docx]

**A traditional evolutionary history of foot-and-mouth disease viruses in Southeast Asia challenged by analyses of non-structural protein coding sequences**

Barbara Brito, Steven J. Pauszek, Ethan J. Hartwig, George R. Smoliga, Le T. Vu, Pham V. Dong, Carolina Stenfeldt, Luis L. Rodriguez, Donald P. King, Nick J. Knowles, Katarzyna Bachanek-Bankowska, Ngo T. Long, Do H. Dung, and Jonathan Arzt

**Supplementary Table 1**

Accession numbers and metadata of viral sequences used for the study. The virus sequences used in the analyses comprise: (1) A total of 29 virus sequences from O/PanAsia collected from the early 2000’ epidemic in China, Taiwan, United Arab Emirates, Japan, South Korea, Malaysia, a sequence collected in China in 2005, and viruses from Vietnam field outbreaks between 2006 and 2010, (2) thirty viral sequences from lineage O/Mya-98 collected from China, Malaysia, Myanmar, Thailand, China, South Korea, Japan, Russia, Mongolia, North Korea, Laos, and Vietnam collected between 2006 and 2014 (3), seventeen sequences classified as serotype A, lineage A/Sea-97 collected from China, Malaysia, South Korea, Thailand, Laos, and Vietnam, collected between 2009 and 2014 (4), eight sequences from O/CATHAY serotype collected between 1997 and 2002 in China, Taiwan and Malaysia. Eight sequences from As1/Gr-V from China, Vietnam and Mongolia between 2005 and 2007, two from As1/Gr-IV collected in Vietnam (2005) and Malaysia (1999), and one from China As1/Gr-VI (2005).

| **Virus name** | **Serotype** | **Lineage/Group** | **Collection date** | **Country** | **Accession number** |
| --- | --- | --- | --- | --- | --- |
| Chu-Pei/1997 | O | CATHAY | 1997 | Taiwan | AF026168 |
| TauYuanTW97 | O | CATHAY | 1997 | Taiwan | AF154271 |
| TWN/1997 | O | CATHAY | 1997 | Taiwan | AF308157 |
| Penghu/iso108/1999 | O | CATHAY | 1999 | Taiwan | AY593833 |
| HK/2001 | O | CATHAY | 2001 | China | EU400597 |
| HKN/2002 | O | CATHAY | 2004 | China | AY317098 |
| YM/YN/2000 | O | CATHAY | 18-Dec-00 | China | HQ412603 |
| MAY/8/2005 | O | CATHAY | 2-Dec-05 | Malaysia | HQ632771 |
| MAY/9/99 | Asia-1 | Group IV | 16-Apr-99 | Malaysia | HQ632774 |
| VN/LC04/2005 | Asia-1 | Group IV | 2005 | Vietnam | GU125646 |
| WHN/CHA/06 | Asia-1 | Group V | 2006 | China | FJ906802 |
| 1/YZ/CHA/06 | Asia-1 | Group V | 26-Jun-06 | China | HQ631363 |
| Jiangsu/China/2005 | Asia-1 | Group V | 2005 | China | EF149009 |
| MOG/05 | Asia-1 | Group V | 2005 | Mongolia | EF614458 |
| HN/2006 | Asia-1 | Group V | 2006 | China | KC412634 |
| HN/CHA/06 | Asia-1 | Group V | 2006 | China | KR073010 |
| Vietnam/QuangTri/2007 | Asia-1 | Group V | 2007 | Vietnam | GQ452295 |
| YS/CHA/05 | Asia-1 | Group V | Aug-05 | China | GU931682 |
| HNK/CHA/05 | Asia-1 | Group VI | 2005 | China | EF149010 |
| GSLX/2010 | O | Mya-98(A) | 2010 | China | JQ900581 |
| MYA/5/2009 | O | Mya-98(A) | 10-Jun-09 | Myanmar | KF112880 |
| VN/LC169/2009 | O | Mya-98(A) | 1-Nov-09 | Viet Nam | HM055510 |
| TAI/22/2009 | O | Mya-98(A) | 18-Nov-09 | Thailand | KF112879 |
| HKN15/2010 | O | Mya-98(A) | 24-Feb-10 | Hong Kong | KF112889 |
| BY/CHA/2010 | O | Mya-98(A) | 1-Mar-10 | China | JN998085 |
| GZ/CHA/2010 | O | Mya-98(A) | 1-Mar-10 | China | JN998086 |
| HKN/20/2010 | O | Mya-98(A) | 3-Mar-10 | Hong Kong | HM229661 |
| SKR/4/2010 | O | Mya-98(A) | 7-Apr-10 | South Korea | KF112886 |
| JPN/1/2010 | O | Mya-98(A) | 17-Apr-10 | Japan | KF112885 |
| RUS/Jul_2010 | O | Mya-98(A) | 5-Jul-10 | Russia | KF112883 |
| SKR/5/2010 | O | Mya-98(A) | 28-Nov-10 | South Korea | KF112887 |
| DRK/31/2011 | O | Mya-98(A) | 1-Jan-11 | North Korea | KF112888 |
| SKR/JC/2014 | O | Mya-98(A) | 30-Jun-14 | South Korea | KX162590 |
| GJ/SKR/2016 | O | Mya-98(A) | 11-Jan-16 | South Korea | KY086465 |
| GC/SKR/2016 | O | Mya-98(A) | 13-Jan-16 | South Korea | KY086466 |
| MAY/3/2014 | O | Mya-98(A) | 25-Feb-14 | Malaysia | KY322672 |
| VN/GL13/2006 | O | Mya-98(B) | 1-Apr-06 | Vietnam | GU125650 |
| VN/SL22/2006 | O | Mya-98(B) | 1-Oct-06 | Vietnam | GU125647 |
| VN/SL21/2006 | O | Mya-98(B) | 1-Oct-06 | Vietnam | GU125649 |
| MAY/7/2007 | O | Mya-98(B) | 20-Oct-07 | Malaysia | HQ632772 |
| VN/YB105/2009 | O | Mya-98(B) | 1-Sep-09 | Vietnam | GU582116 |
| VN/QB88/2009 | O | Mya-98(B) | 1-Oct-09 | Vietnam | GU582115 |
| VIT/19/2010 | O | Mya-98(B) | 22-Jan-10 | Vietnam | MF947135 |
| MOG/C-10/2010 | O | Mya-98(B) | 1-May-10 | Mongolia | KF112882 |
| MOG/7/2010 | O | Mya-98(B) | 6-Sep-10 | Mongolia | KF112881 |
| LAO/1/2013 | O | Mya-98(B) | 19-Jul-13 | Laos | KY322670 |
| MAY/2/2014 | O | Mya-98(B) | 6-Feb-14 | Malaysia | KY322671 |
| MAY/8/2014 | O | Mya-98(B) | 10-Jun-14 | Malaysia | KY322673 |
| VIT/11381/2014 | O | Mya-98(B) | 14-Jul-14 | Vietnam | MF947130 |
| China/1/99Tibet | O | PanAsia | 1999 | China | AF506822 |
| TAW/2/99 BOV | O | PanAsia | 1999 | Taiwan | AJ539137 |
| UAE 7/99 | O | PanAsia | 1999 | United Arab Emirates | EU140964 |
| JPN/2000 | O | PanAsia | 2000 | Japan | AB079061 |
| SKR/2000 | O | PanAsia | 2000 | South Korea | AF377945 |
| SKR/2000 | O | PanAsia | 2000 | South Korea | AJ539139 |
| SAR/19/2000 | O | PanAsia | 2000 | South Africa | AJ539140 |
| UKG/2000/2001 | O | PanAsia | 2001 | United Kingdom | FJ542369 |
| MAY/3/2000 | O | PanAsia | 11-Feb-00 | Malaysia | HQ632768 |
| S15KOR/2002 | O | PanAsia | 10-Jun-02 | South Korea | KF694745 |
| YS/CHA/05 | O | PanAsia | 1-Oct-05 | China | HM008917 |
| VIT/7223/2010 | O | PanAsia | 1-Oct-10 | Vietnam | MF947128 |
| VIT/169/2010 | O | PanAsia | 30-Nov-10 | Vietnam | MF947138 |
| VIT/190/2010 | O | PanAsia | 6-Dec-10 | Vietnam | MF947139 |
| VIT/217/2010 | O | PanAsia | 9-Dec-10 | Vietnam | MF947140 |
| VIT/20/2011 | O | PanAsia | 14-Jan-11 | Viet Nam | MF947123 |
| VIT/89/2011 | O | PanAsia | 16-Feb-11 | Vietnam | MF947125 |
| VIT/1677/2011 | O | PanAsia | 22-Feb-11 | Viet Nam | MF947126 |
| VIT/30/2012 | O | PanAsia | 11-Apr-12 | Viet Nam | MF947124 |
| VIT/3910/2012 | O | PanAsia | 16-Apr-12 | Vietnam | MF947141 |
| VIT/12/2012_pro | O | PanAsia | 9-May-12 | Vietnam | MF947134 |
| VIT/20/2012_pro | O | PanAsia | 23-May-12 | Vietnam | MF947136 |
| VIT/2012/Buff(V-BU1)/Phar(A) | O | PanAsia | 5-Nov-12 | Viet Nam | MF947127 |
| VIT/49/2012_pro | O | PanAsia | 5-Nov-12 | Vietnam | MF947137 |
| VIT/106131/2013 | O | PanAsia | 17-Aug-13 | Vietnam | MF947143 |
| VIT/10895KV1/2013 | O | PanAsia | 24-Aug-13 | Vietnam | MF947142 |
| VIT/11965/2013 | O | PanAsia | 9-Oct-13 | Vietnam | MF947131 |
| VIT/10894/2014 | O | PanAsia | 10-Jul-14 | Vietnam | MF947129 |
| VIT/16456/2015 | O | PanAsia | 10-Sep-15 | Vietnam | MF947132 |
| VIT_4/2004 | A | Sea-97 | 2004 | Vietnam | HQ268509 |
| VN/T11D/2013 | A | Sea-97 | 5-Jul-05 | Vietnam | KJ608371 |
| MAY/3/2007 | A | Sea-97 | 22-Jul-07 | Malaysia | HQ632773 |
| VN/03/2009 | A | Sea-97 | 9-Jan-09 | Vietnam | GQ406249 |
| VN/09/2009 | A | Sea-97 | 6-Feb-09 | Vietnam | GQ406247 |
| VN/11/2009 | A | Sea-97 | 6-Feb-09 | Vietnam | GQ406250 |
| VN/16/2009 | A | Sea-97 | 17-Feb-09 | Vietnam | GQ406251 |
| VN/20/2009 | A | Sea-97 | 19-Feb-09 | Vietnam | GQ406252 |
| Pocheon/001/KOR/2010 | A | Sea-97 | 7-Jan-10 | South Korea | KC588943 |
| VIT/12/2010 | A | Sea-97 | 15-Jan-10 | Vietnam | MF947133 |
| HY/CHA/2013 | A | Sea-97 | 15-Aug-13 | China | KT968663 |
| MAY/12/2013 | A | Sea-97 | 12-Oct-13 | Malaysia | KY322676 |
| VIT/42/2013 | A | Sea-97 | 16-Oct-13 | Vietnam | KY322680 |
| MAY/20/2013 | A | Sea-97 | 25-Nov-13 | Malaysia | KY322677 |
| MAY/23/2013 | A | Sea-97 | 26-Dec-13 | Malaysia | KY322678 |
| TAI/4/2014 | A | Sea-97 | 16-Jan-14 | Thailand | KY322679 |
| LAO/3/2014 | A | Sea-97 | 20-Feb-14 | Laos | KY322675 |
